# Gastric Carcinomas Localized to the Cardia

**DOI:** 10.1155/2012/457831

**Published:** 2012-02-28

**Authors:** Karin E. Bakkelund, Ivar S. Nordrum, Reidar Fossmark, Helge L. Waldum

**Affiliations:** ^1^Department of Cancer Research and Molecular Medicine, Faculty of Medicine, NTNU, 7006 Trondheim, Norway; ^2^Department of Laboratory Medicine, Children's and Women's Health, Faculty of Medicine, NTNU, 7006 Trondheim, Norway; ^3^Department of Pathology and Medical Genetics, St. Olavs Hospital, 7006 Trondheim, Norway; ^4^Department of Gastroenterology and Hepatology, St. Olavs Hospital, 7006 Trondheim, Norway

## Abstract

*Objectives*. Gastric adenocarcinomas localized to the cardia are increasing. Enterochromaffin-like (ECL) cells play a role in gastric carcinogenesis in hypergastrinemia, and the use of proton pump inhibitors (PPI) leading to hypergastrinemia has increased considerably during the last decades. We have examined cardia cancers for neuroendocrine and ECL cell differentiation. *Methods*. Thirty-two cardia cancers were examined by immunohistochemical labelling of chromogranin A (CgA), synaptophysin, serotonin, and histidine decarboxylase (HDC). Information about PPI use was collected from the patient records. *Results*. In 15 of 32 tumours, there were positive signs for one or several neuroendocrine markers. Five cases were CgA and serotonin positive; three of these carcinomas were also positive for HDC. Three patients were long-term users of PPI, and two of these were immunoreactive for neuroendocrine markers. *Conclusions*. A high proportion of cardia cancers expressed neuroendocrine markers, but only few patients with cardia cancers were using PPI.

## 1. Introduction

Gastric adenocarcinoma has shown a marked decline in the last decades [1]. Nevertheless, there seems to be an increase in carcinomas localized to the cardia [2–5], especially in younger women and older men [6]. However, other studies have failed to demonstrate such an increase [7–9]. 

Little is known about the mechanisms for the development of cardia cancers. Adenocarcinomas of the esophagus and cardia share some epidemiological features, including incidence trends and sex distribution [9], but could have different risk factors. Oesophageal carcinoma is associated with reflux disease, obesity, hiatal hernia, and smoking, but these factors may have weak or no association with cardia cancer [10–13]. 

*H. pylori* infection is associated with cancers distal to cardia, but in cardia cancers there seems to be a negative association [14]. Hypergastrinemia is associated with noncardia cancers, but not with cardia cancers in general. However, in *H. Pylori-*positive persons, there may be an association [14]. 

Neuroendocrine cells comprise about 1% of the volume of the gastric oxyntic mucosa [15], but little is known about the presence of neuroendocrine cells in the cardia. One study indicates that neuroendocrine cells in the normal cardia stain positive for serotonin only [16], occurring in an inflamed cardiac mucosa independent of intestinal metaplasia, a well-known risk factor for cardia cancer [17]. However, the presence of enterochromaffin-like (ECL) cells was not examined.

We have repeatedly shown that the ECL cell plays an important role in gastric carcinogenesis [18–21]. The growth of the ECL cell is mainly regulated by gastrin, which in turn reflects gastric acidity. Since the use of efficient inhibitors of gastric acid secretion leading to hypoacidity and hypergastrinemia has increased dramatically during the last decades, we wanted to retrospectively examine gastric cardiac cancers for neuroendocrine and particularly ECL cell differentiation.

## 2. Material and Methods

Thirty-two gastric carcinomas located to the cardia or less than 1.5 cm from oesophagus border were included consecutively from the files of the Department of Pathology and Medical Genetics, St. Olavs Hospital. Twenty-six cases were males (mean age 65.5 years) and six females (mean age 69.2 years).  Table 1 shows the characteristics of the tumours. The patient files were examined for use of proton pump inhibitors (PPI), and we found long-term use (ten years or more) in three patients.

Serial sections 4 *μ*m thick were cut from formalin-fixed paraffin-embedded tissue and deparaffinised. Sections for morphology were stained for hematoxylin and eosin. Sections for immunohistochemical labelling were immersed in 3% hydrogenperoxide to block endogenous peroxidase activity. Antigen retrieval before immunolabelling was achieved by boiling the sections in Tris/EDTA-buffer pH 9.0 (synaptophysin, histidin decarboxylase (HDC), and serotonin) or citrate-buffer pH 6.0 (Chromogranin A (CgA)) for 15 minutes. The sections were incubated with antibody against synaptophysin (Code A010, Dakocytomation, Glostrup, Denmark, 1 : 200) and HDC (Code B260-1, Eurodiagnostica, 1 : 15000) for 1 hour at room temperature, and CgA (Code MO869, Dakocytomation, 1 : 2000) and serotonin (Code 6336, Abcam, 1 : 100) for 18 hours at 4°C. Tyramide signal amplification was used to increase sensitivity of CgA labelling as described previously [19].

Antigen-antibody complexes were visualized using the Envision-HRP kit (K5007, Dakocytomation) and AEC (SK4200, Vector laboratories Burlingame, CA) or DAB+ (K5007, Dakocytomation). Finally, the sections were counterstained with haematoxylin.

Differences between groups were evaluated using two-tailed Mann-Whitney *U* test when comparing. *P* values < 0.05 were considered statistically significant. Statistical analyses were done using Graphpad Prism 4.0 (Graphpad Software Inc., San Diego, CA).

The study was approved by The Regional Committee for Medical Research Ethics in Trondheim, Norway.

## 3. Results

According to Laurens classification, 23 of the 32 tumours were classified as intestinal type, five as diffuse type, three of mixed type, and one undetermined (Table 1).

There was a male predominance: 26 male and 6 female patients.

**Figure 1:**
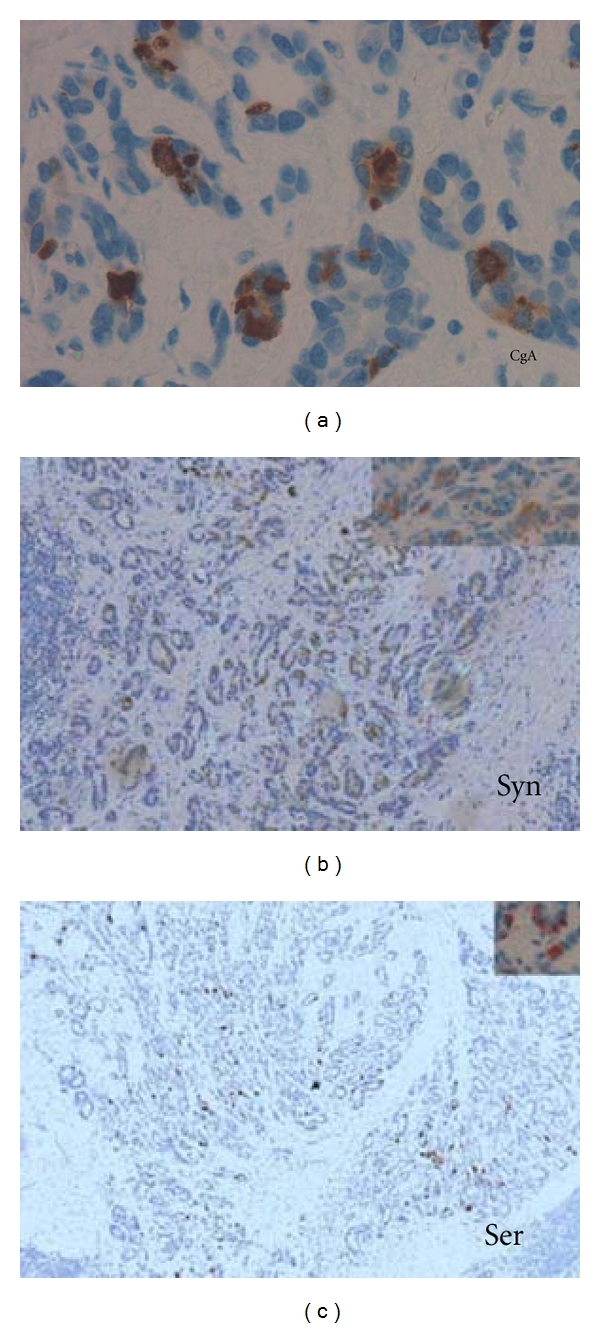
1 Immunohistochemical labelling of tumour cells in the gastric cardiac region in patient 4. Tumour cells label positive for CgA (40x) (a), synaptophysin (b), and serotonin (c) 20x. Inserts 40x.

**Figure 2:**
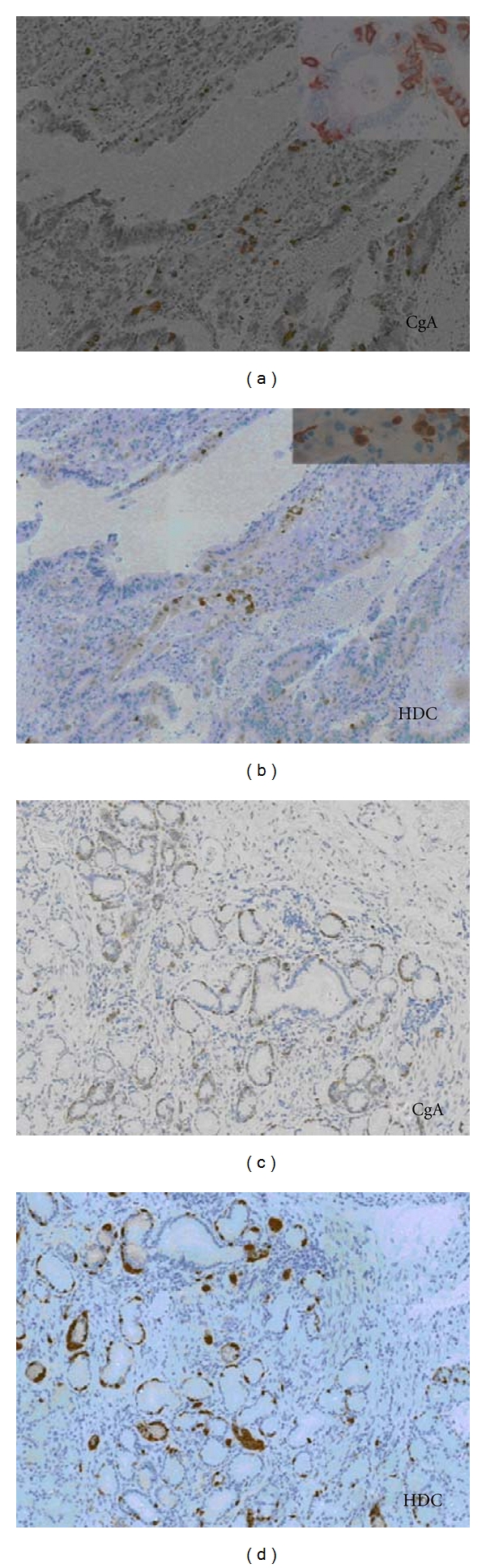
Immunohistochemical labelling of tumour cells in the gastric cardiac region in patient 9 demonstrating positive labelling of CgA (a and c) and HDC (b and d) (20x). Inserts 40x.

In 15 of the 32 tumours, some tumour cells were positive for one or more neuroendocrine markers, CgA and serotonin in five cases. Three of these were also positive for HDC (Figure 1). Seven were positive for one marker only, synaptophysin most predominantly (Table 2). One patient had linear hyperplasia and micronodules positive for HDC and CgA in mucosa adjacent to the tumour (Figure 2). HDC-and serotonin-positive cells were present in the normal gastric mucosa. There was a nonsignificant tendency that female patients more often had neuroendocrine differentiation (5 of 6 women versus 9 of 26 men, *P* = 0.07). 

There was no difference in frequency of neuroendocrine differentiation between diffuse and intestinal cancers (4 of 8 diffuse versus 10 of 24 intestinal, *P* = 0.74).

There was no difference in age between patients with neuroendocrine labelling or without neuroendocrine staining (67 ± 3 years versus  68 ± 2 years). Three patients reported use of PPI, and two of these had neuroendocrine labelling of tumour cells. In the normal-appearing mucosa outside the carcinomas, there were cells positive for HDC in most patients, indicating that ECL cells are present in the normal cardia.

## 4. Discussion

Gastric carcinomas in the cardiac region have shown an unexplained increase in frequency [2–6], and it was of interest to see if this increase could be related to use of PPI. Carcinomas developing in hypergastrinemic patients are immunoreactive for neuroendocrine markers, and we therefore examined cardia cancers for such markers.

In rats, gastric cancers develop from the ECL-cells after long-term treatment with omeprazole, a potent PPI [22]. Nevertheless, omeprazole and later other PPIs were accepted for clinical use based upon the assumption that ECL-cell- derived gastric tumours were rare or insignificant in man. On the other hand, although PPI treatment results in ECL cell hyperplasia [23], only one indirect report on ECL cell carcinoids during treatment with PPI exists [24]. Moreover, after extensive use of PPIs in Western countries for at least fifteen years, there has been no report confirming an association between the increase in gastric carcinomas and PPI use. However, there is an absolute increase in gastric carcinomas of diffuse type, particularly of signet-ring cell subtype [25] among which ECL-cell-derived carcinomas were most often found [18, 21, 26, 27]. There is also an increase in noncardia gastric carcinomas in the younger age-groups in the USA [28].

An epidemiological study found an association between use of PPI and oesophageal carcinomas and noncardia carcinomas, but not with cardiac carcinomas [29]. The major limitation of that study was the short medication time (mean 3 years), and the findings could be a result of the foregoing disease and not the medication. Our study is a relatively small study but larger prospective studies of patients using long-term PPI have so far not been published. We found that only three of thirty-two patients with gastric cardiac carcinoma had been using PPI for more than 10 years. A significant association between PPI and cardiac carcinoma could not be detected in this study, and this study does not support the hypothesis that use of inhibitors of acid secretion could explain the increased occurrence of gastric carcinomas localized to the cardia.

Human gastric carcinomas are divided into two types according to Lauren: diffuse and intestinal [30], with different epidemiology, risk factors, and cell of origin [18]. Twenty-three of the thirty-two patients had an intestinal type, and five had a diffuse type. Three were classified as a mixed type. Other studies have also reported that cardia cancers are predominantly of intestinal type [14, 31], whereas ECL-cell-derived cancer developing in hypergastrinemic patients with pernicious anaemia are of the diffuse type [19]. 

In the present study, fifteen of thirty-three cases of gastric cardiac cancer expressed neuroendocrine markers, ten of these were classified as intestinal type, five were diffuse, and one had a mixed growth pattern. This does not lend support to the hypothesis that ECL-cell-derived tumours develop in gastric cardia.

Three patients reported use of PPI, and two of these had neuroendocrine staining of the tumour cells. These tumours were serotonin immunoreactive. The mechanisms involved in growth regulation of EC cells are not known, but there are no studies supporting that gastrin acts as a growth factor for EC cells.

However, the serotonin-positive tumours also stained weakly positive for HDC, an ECL cell marker. One possible explanation is that EC cells and ECL cells are closely related, and during malignant transformation, the EC cells could express ECL cell markers and vice versa.

The existence of a normal cardiac mucosa has been questioned, and the physiological role of this tissue is unknown. The mucosa considered to be normal in cardia could be a metaplastic area that develops as a result of gastroesophageal reflux disease [32, 33], a view that has been contradicted by others [34]. Distal extension of cardia could arise from atrophic gastritis, most commonly induced by *H. pylori* infection. 

The neuroendocrine cells in cardia are not well characterized. The extracardiac gastric mucosa contains various neuroendocrine cells such as ECL cells, G cells, A-like cells, and D cells, while the cardia is believed to contain only serotonin-producing cells, i.e., EC cells [16]. However, ECL cells, which are the most abundant neuroendocrine cell in the oxyntic mucosa, were not examined for in this study. We have found that ECL cells are present in the gastric cardiac mucosa outside the tumours, indicating that the cardiac region resembles the oxyntic mucosa as regards the neuroendocrine cell population. 

In conclusion, almost half the cardia cancers expressed neuroendocrine markers, but few patients had been long-term PPI users. The present study does not provide evidence that use of inhibitors of acid secretion could explain the increased occurrence of gastric carcinomas localized to the cardia. 

## Figures and Tables

**Table 1:** Characteristics of gastric cardiac carcinomas.

Case no.	Tumour diameter (cm)	Histological grading	Lauren classification	Depht of infiltration	Lymph nodes with metastasis
1	3	Md	Int	Ss	6/7
2	4	Muc	Int	Mp	3/12
3	8	Pd	Diff	Ss	1/9
4	3,2	Pd	Mix	Ss	0/12
5	3	Pd	Int	Mp	10/12
6	6	Pd	Mix	Ss	2/12
7	4	Pd	Int	Ss	6/18
8	4,5	Md	Int	Ss	5/8
9	4	Pd	Int	Ss	10/10
10	1,2	Md	Int	Ss	0/6
11	3,2	Hd	Int	Mp	6/11
12	4	Muc	Mix	Ss	3/5
13	4,5	Pd	Int	Ss	2/12
14	9	Pd	Int	Ss	8/12
15	4,5	Md	Int	Ss	2/11
16	5	Pd	Int	Ss	3/5
17	5	Md	Int	Ss	0/2
18	6	Md	Int	Ss	12/16
19	3,0	Pd	Diff	Ss	3/9
20	4,6	Pd	Int	Ss	7/11
21	4,2	Md	Int	Mp	0/11
22	2,2	Md	Int	Ss	2/2
23	5,0	Pd	Diff	Ss	2/16
24	4,0	Pd	Int	Ss	0/1
25	2,5	Pd	Diff	Ss	1/9
26	4,0	Ld	Int	Ss	12/14
27	5,0	Ld	Undetermined	Ss	3/15
28	4,8	Md	Int	Ss	2/3
29	2,0	Pd	Diff	Ss	5/7
30	9,0	Md	Int	Ss	1/6
31	5,7	Md	Int	Ss	0/3
32	4,0	Ld	Int	Ss	2/13

Pd: Poorly differentiated, Md: Moderately differentiated, Hd: Highly differentiated, Muc: Mucinous, Int: Intestinal, Diff: Diffuse, Mix: Mixed type, Mp: The tumour grows into but not through muscularis propria, Ss: The tumour grows through muscularis propria.

**Table 2:** Neuroendocrine differentiation in gastric carcinomas localized to the cardia and long-term use of proton pump inhibitors.

Case no.	CgA	Synaptophysin	HDC	Serotonin	Use of PPI
1	−	−	−	−	−
2	−	−	−	−	−
3	−	−	−	−	−
4	+	+	−	+	−
5	+	−	−	+	+
6	−	−	−	−	−
7	−	−	−	−	−
8	−	−	−	−	−
9	+	−	+	+	−
10	−	−	−	−	−
11	+	+	+	+	+
12	−	−	−	−	−
13	−	−	−	−	+
14	−	−	−	−	−
15	−	−	−	−	−
16	−	−	−	−	−
17	−	−	−	−	−
18	+	−	−	−	−
19	+	−	−	−	−
20	+	+	−	−	−
21	−	+	−	−	−
22	−	+	−	−	−
23	−	+	−	−	−
24	+	+	−	−	−
25	−	+	−	−	−
26	−	−	−	−	−
27	−	−	−	−	−
28	+	−	−	−	−
29	+	+	−	−	−
30	−	−	−	−	−
31	+	+	+	+	−
32	−	−	−	−	−
